# Quality-of-life measures and their psychometric properties used in African chronic kidney disease populations: a systematic review using COSMIN methodology

**DOI:** 10.1186/s12882-024-03482-5

**Published:** 2024-02-09

**Authors:** Moustapha Faye, Florian Manneville, Adama Faye, Luc Frimat, Francis Guillemin

**Affiliations:** 1 Service de Néphrologie, CHU Aristide Le Dantec, Université Cheikh Anta Diop, Dakar, Sénégal; 2 Université de Lorraine, APEMAC, Nancy, France; 3 CHRU-Nancy, INSERM, Université de Lorraine, CIC Epidémiologie Clinique, Nancy, 54000 France; 4 Institut Santé Et Développement (ISED), Université Cheikh Anta Diop de Dakar, Dakar, Senegal; 5 Service de Néphrologie, CHRU-Nancy Brabois Santé, Vandœuvre-lès-Nancy, France

**Keywords:** Quality of life, Psychometric, Chronic kidney disease, Africa, Systematic review

## Abstract

**Background:**

If any benefit is to be derived from the use of the health-related quality of life (HRQoL) questionnaires in chronic kidney disease (CKD) patients, they should be validated and culturally adapted to the target population. We aimed to critically appraise the psychometric properties of HRQoL questionnaires used in African populations with CKD.

**Methods:**

Web of Science, Embase, PubMed and PsycINFO databases were searched. Psychometric validation studies of HRQoL questionnaires reporting at least one psychometric property of the COSMIN checklist in CKD African population, published up to October 16, 2023 were included and independently assessed for methodological quality and level of measurement properties by using the COSMIN methodology.

**Results:**

From 1163 articles, 5 full-text were included. Only the Kidney Disease Quality-of-Life questionnaire was translated and cross-culturally adapted for studies of patients with CKD. Internal consistency was of doubtful quality in 4 studies and very good in 1. Its measurement was sufficient in 1 study and insufficient in 4. Test–retest reliability was of doubtful quality in 4 studies. Its measurement was sufficient in 3 studies and insufficient in 1. Structural validity was of inadequate quality in 1 study and very good quality in 1. Its measurement was sufficient in both. Construct validity was of inadequate quality in all studies. Their measurement was insufficient in 4 studies and sufficient in 1.

**Conclusions:**

This review highlighted that only one HRQoL questionnaire used in studies of African populations with CKD underwent a small number of cultural adaptations and psychometric validations, generally of poor methodological quality. HRQoL validation studies in African CKD populations are needed to better take advantage of the benefits in patient care, population health management, and research.

**Supplementary Information:**

The online version contains supplementary material available at 10.1186/s12882-024-03482-5.

## Introduction

Chronic kidney disease (CKD) is a global public health problem because of its prevalence, severity [1], complexity [2] and high cost of management [3, 4]. The incidence and prevalence of CKD has been increasing over the years [5]. Africa is affected by the double burden of infectious diseases and chronic non-communicable diseases including CKD [6, 7].

Health-related quality of life (HRQoL), a multidimensional concept, is an important clinical endpoint for patients, healthcare providers, and funding partners [8, 9]. CKD patients have to deal with significant lifestyle changes that affect their HRQoL in all its dimensions. HRQoL is negatively affected by CKD glomerular filtration category G3 to G5, with or without kidney replacement therapy [10]. HRQoL is worse in the CKD population than in the general population [10, 11]. The physical component score of an HRQoL survey (0–100 scale) was found to be 42.6, 40.3, and 34.8, respectively (the higher the score, the better the quality of life), in patients with moderate CKD, in those with advanced CKD, and in those on dialysis [10].

HRQoL and symptoms (a dimension of HRQoL) can be assessed using validated, self-administered questionnaires characterized as patient-reported outcome measures (PROMs) [12]. These instruments strongly depend on the sociocultural context of the target population. Thus, they must be culturally adapted following published guidelines [13] and validated on the basis of classical or modern psychometric properties. Several instruments measure HRQoL in CKD. Some are generic, such as the Medical Outcome Survey 36-item Short Form [14], the Nottingham Health Profile [15], the World Health Organization Quality of Life [16]. Others are specific to kidney disease, such as the Kidney Disease Quality of Life (KDQOL) [17, 18], the Kidney Disease Questionnaire [19], the CHOICE Health Experience Questionnaire [20], the Dialysis Symptom Index [21], the Modified Edmonton Symptom Assessment System [22], and the End-Stage Renal Disease Symptom Checklist-Transplant Module [23].

If any benefit is to be derived from the use of these questionnaires, they should be validated and culturally adapted to the target population.

The COnsensus-based Standards for the selection of health status Measurement INstruments (COSMIN) is a methodological quality assessment of psychometric studies [24]. COSMIN recommends that 3 quality domains of HRQoL instruments (reliability, validity, and responsiveness) be assessed for systematic reviews [24].

There are few systematic reviews of the psychometric properties of HRQoL assessment, and they include few studies conducted in Africa. Two systematic reviews [17, 18] of the psychometric properties of HRQoL assessment worldwide included only 2 African studies [25, 26]. Several studies of the assessment of HRQoL in CKD have been conducted in the African continent, sometimes in areas where the KDQOL is not culturally adapted, such as Senegal [27, 28]. To our knowledge, no systematic review of the psychometric properties of HRQoL assessment has been conducted in Africa. A systematic review targeting exclusively African literature, including a manual search of African researchers can enable to increase the sensitivity of the detection of validation studies. We hypothesize that few psychometric validation studies of HRQoL questionnaires in CKD have been conducted in Africa despite the use of these questionnaires in HRQoL assessment in Africa CKD population.

The aim of the present systematic review was to identify, critically appraise and summarize the psychometric properties of instruments measuring HRQoL in CKD and their cross-cultural adaptation in African populations living with CKD using the COSMIN methodology.

## Methods

### Study design

This was a systematic review using the COSMIN recommendations for systematic reviews of PROMs [29] and the COSMIN methodology for assessing the quality of psychometric studies [24]. The results are reported according to the Preferred Reporting Items for Systematic Reviews and Meta-Analysis (PRISMA) [30] (see Supplementary file 1: Additional file 1). The review protocol was prepared but has not been registered beforehand.

### Search methods

The following databases were searched for the literature review: Web of Science, Embase, PubMed/MEDLINE, PsycINFO. A manual search was also performed using the references of the various articles or with fellow African researchers through social media (Twitter, WhatsApp). The search was limited to all articles in French and English and published up to October16, 2023. The search equations were constructed from the following keywords: construct, population, instrument name, and psychometric properties. For the PubMed and Embase search, the PubMed filters for searching psychometric studies were used [31] (see Supplementary file 1: Additional file 2).

### Eligibility criteria

Studies were selected according to the following criteria: Type of participant: African population living with CKD G1 to G5, dialysis patients or kidney transplant recipients; measurement instrument: any measurement instrument for HRQoL in CKD; type of study: psychometric validation studies reporting at least one psychometric property of the COSMIN checklist, and published in English or French. We excluded conference or congress abstracts, editorials, clinical cases, reviews, theses and commentaries.

### Selection of the studies

One author (MF) removed duplicates and selected articles on the basis of titles and abstracts by using the Rayyan tools website (https://www.rayyan.ai/). Then 2 independent authors (MF, FG) evaluated the full text and resolved discrepancies by consensus.

### Methodological quality assessment of the included studies

Two independent authors (MF, FG) assessed the quality of the included studies using the COSMIN Risk of Bias assessment checklist [24]. The 2 authors discussed discrepancies to reach consensus. The checklist included 10 boxes for content (development of PROMs, content validity), internal structure (structural validity, internal consistency, cross-cultural validity/invariance of the measure), and measurement properties (reproducibility, measurement error, criterion validity, hypothesis testing for construct validity, responsiveness). The process of translating the questionnaire was evaluated (back translation, expert committee, cognitive debriefing). Each study was scored as “very good quality “, “adequate quality “, “doubtful quality “, or “inadequate quality “.

### Measurement property assessment of each study

The psychometric properties for each study were scored on the basis of criteria for good measurement properties (Table 1) [29]. Each score was reported as sufficient (+), insufficient (-), or indeterminate (?) and was evaluated by 2 authors (MF, FG). The following properties were assessed: reliability (internal consistency, test–retest reliability); validity (content validity, construct validity, cross-cultural validity/measurement invariance, measurement error, criterion validity, hypothesis testing for construct validity); and responsiveness.

**Table 1:** Criteria for good measurement properties according to the checklist [29]

Measurement property	Rating^a^	Criteria
Structural validity	**+ **	**CTT:** CFA: CFI or TLI or comparable measure > 0.95 OR RMSEA < 0.06 OR SRMR < 0.08^b^ **IRT/Rasch**: No violation of unidimensionality^c^: CFI or TLI or comparable measure > 0.95 OR RMSEA < 0.06 OR SRMR < 0.08 *AND* no violation of local independence: residual correlations among the items after controlling for the dominant factor < 0.20 OR Q3’s < 0.37 *AND* no violation of monotonicity: adequate looking graphs OR item scalability > 0.30 *AND* adequate model fit: IRT: χ^b^ > 0.01 Rasch: infit and outfit mean squares ≥ 0.5 and ≤ 1.5 OR Z‐ standardized values > ‐2 and < 2
	**?**	CTT: Not all information for ‘ + ’ reported IRT/Rasch: Model fit not reported
	**-**	Criteria for ‘ + ’ not met
Internal consistency	**+ **	Criteria for “At least low evidence^d^ for sufficient structural validity^e^ “ not met
	**?**	Criteria for “At least low evidence^d^ for sufficient structural validity^e^ “ not met
	**-**	At least low evidence^d^ for sufficient structural validity^e^ AND Cronbach’s alpha(s) < 0.70 for each unidimensional scale or subscale^f^
Reliability	**+ **	ICC or weighted Kappa ≥ 0.70
	**?**	ICC or weighted Kappa not reported
	**-**	ICC or weighted Kappa < 0.70
Measurement error	**+ **	SDC or LoA < MIC^e^
	**?**	MIC not defined
	**-**	SDC or LoA > MIC^e^
Hypotheses testing for construct validity	**+ **	The result is in accordance with the hypothesis^g^
	**?**	No hypothesis defined (by the review team) The result is not in accordance with the hypothesis^g^
	**-**	The result is in accordance with the hypothesis^g^
Cross‐cultural validity\measurement invariance	**+ **	No important differences found between group factors (such as age, gender, language) in multiple group factor analysis OR no important DIF for group factors (McFadden’s R^b^ < 0.02)
	**?**	No multiple group factor analysis OR DIF analysis performed
	**-**	Important differences between group factors OR DIF was found
Criterion validity	**+ **	Correlation with gold standard ≥ 0.70 OR AUC ≥ 0.70
	**?**	Not all information for ‘ + ’ reported Correlation with gold standard < 0.70 OR AUC < 0.70
	**-**	Correlation with gold standard ≥ 0.70 OR AUC ≥ 0.70
Responsiveness	**+ **	The result is in accordance with the hypothesis^g^ OR AUC ≥ 0.70
	**?**	No hypothesis defined (by the review team)
	**-**	The result is not in accordance with hypothesis^g^ or AUC < 0.70

*AUC* area under the receiver operating characteristic curve, *CFA* confirmatory factor analysis, *CFI* comparative fit index, *CTT* classical test theory, *DIF* differential item functioning, *ICC* intraclass correlation coefficient, *IRT* item response theory, *LoA* limits of agreement, MIC minimal important change, *RMSEA* root mean square error of approximation, *SEM* standard error of measurement, *SDC* smallest detectable change, *SRMR* standardized root mean residuals, *TLI* Tucker‐Lewis index

^a^“ + “ = sufficient, “ – “ = insufficient, “? “ = indeterminate

^b^To rate the quality of the summary score, the factor structures should be equal across studies

^c^unidimensionality refers to a factor analysis per subscale, and structural validity refers to a factor analysis of a (multidimensional) patient‐reported outcome measure

^d^As defined by grading the evidence according to the GRADE approach

^e^This evidence may come from different studies

^f^The criterion Cronbach alpha < 0.95 was deleted because this is relevant in the development phase of a PROM but not when evaluating an existing PROM

^g^The results of all studies should be taken together and then decided if 75% of the results are in accordance with the hypotheses

### Data extraction and synthesis

One author (MF) extracted data by using a predefined Excel sheet. The extracted data related to the population (sample size, sex, age, target population) and characteristics of the questionnaire (type of administration, language, country).

The synthesis of the psychometric properties of the studies was performed in accordance with COSMIN recommendations [24, 29, 32].

## Results

### Description of the included studies

**Fig. 1:**
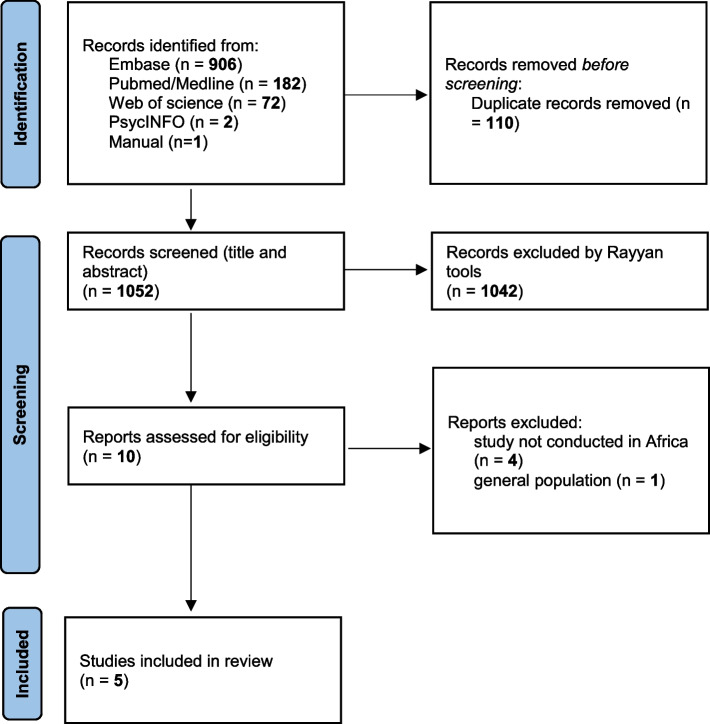
PRISMA flow chart diagram

As a result of the literature search, 1163 articles were screened. After removing duplicates, 1052 articles were evaluated for relevance according to the title and/or the abstract. Ten full-text articles were screened, and finally 5 full-text articles were included in the review. The PRISMA flow chart of the study selection is presented in Fig. 1.

The 5 studies evaluated only one questionnaire, the KDQOL. It was validated in all studies and applied to patients with CKD G3 to G5 who were undergoing dialysis (haemodialysis or peritoneal dialysis) or who had renal transplantation. The sample sizes of the 5 studies ranged from 80 to 363 (Table 2). Two studies reported a mean (± standard deviation) age of 43.9 ± 14.2 and 48 ± 14.7 years [26, 33]; 2 others reported a median (interquartile range) age of 46 (35–58) and 54 (42–60) [34, 35]. Among 3 studies, the proportion of males ranged from 54 to 64% [33–35]. One study reported a predominance of women [26]. The studies were conducted in Morocco, Egypt, Ethiopia, Sudan, and Uganda. The original English version of KDQOL was translated into Arabic, Amharic, and Luganda. Backward translation and expert committee were used in all studies and cognitive debriefing in 4 [26, 33–35].

**Table 2:** Characteristics of the included studies

**Author, year of publication**	**Bouidida et al. 2014 **[26]	**Abd ElHafeez et al. 2012 **[34]	**Gebrie et al. 2022 **[33]	**Elamin et al. 2019 **[25]	**Bagasha et al. 2022 **[35]
KDQOL (item number)	79	36	36	36	79
Country	Morocco	Egypt	Ethiopia	Sudan	Uganda
Sample	80	100	292	144	364
Population	HD = 62; PD = 18	CKD at G3-G4	HD	HD = 62; KT = 82	CKD G5 in CC or HD
Men (%) or sex ratio	0.7	54	64	NR	60
Age (years)^a^	43.9 (14.2)	54 (42–60)	48 ± 14.7	NR	46 (35–58)
Literacy (%)	28	24	26	NR	NR
Cognitive debriefing	Yes	Yes	Yes	No	Yes
Language	Morocco (Arabic)	Arabic	Amharic	Arabic	Luganda
Backward translation	Yes	Yes	Yes	Yes	Yes
Expert committee	Yes	Yes	Yes	Yes	Yes

*KDQOL* kidney disease quality of life, *HD* haemodialysis, *PD* peritoneal dialysis, *CKD* chronic kidney disease, *KT* kidney transplant, *CC* conservative care, *NR* not reported

^a^mean ± SD or median (interquartile range)

### Methodological quality of the included studies

The methodological quality of each study is presented in Table 3. No study reported PROM development, measurement error, cross-cultural validity, criterion validity, or responsiveness.

**Table 3:** Methodological quality of the included studies

Author, year of publication	Structural validity	Internal consistency	Test–retest reliability	Hypotheses testing for construct validity
Bouidida et al. 2014 [26]	NR	Doubtful	Doubtful	Inadequate
Abd ElHafeez et al. 2012 [34]	Inadequate	Inadequate	Doubtful	Inadequate
Gebrie et al. 2022 [33]	Very good	Very good	Doubtful	Inadequate
Elamin et al. 2019 [25]	NR	Doubtful	Doubtful	Inadequate
Bagasha et al. 2022 [35]	NR	Doubtful	NR	Inadequate

Patient reported outcomes measure (PROM) development, Measurement error, cross-cultural validity, criterion validity, and responsiveness were not reported in any of the studies

*NR* not reported

### Reliability

*Internal consistency* was assessed in all 5 studies. It was rated as very good in one study [33], inadequate in one study [33] and doubtful in others because structural validity (unidimensionality of scales or sub-scales) was not reported in these studies.

*Test–retest reliability* was assessed in 4 studies [25, 26, 33, 34]. It was rated doubtful in all studies because the article did not clarify whether patients were stable or what was the time interval used.

The intraclass correlation (ICC) was calculated, but the model and formula were not described in 2 studies [26, 33] and the ICC model was derived from a two-way random effects model in 1 study [25]. The time interval was 10 to 14 days in 2 study [26], and 2 weeks in 2 studies [25, 34].

### Validity

*Structural validity* was assessed in 2 studies. It was rated inadequate in one study [34] because the sample size included fewer than 5 times the number of items, and very good in one study [33].

*Hypothesis testing for construct validity *was assessed in all studies. Known group validity was assessed in all studies and convergent validity in 4 [25, 26, 33, 34]. Hypothesis testing for construct validity was rated inadequate in 4 studies [25, 26, 34, 35] because the comparator had insufficient measurement properties or no information on these measurement properties. These studies used the physical component and mental component scores of the Arabic version of KDQOL-36 to be validated, the Arabic version of the Depression Anxiety Stress Scale-21 (DASS-21), and overall health rate as comparator. It was rated inadequate in 1 study [33] because the correlation of the score with that of the comparator instrument (5-level EuroQol 5-dimensional questionnaire [EQ-5D-5L] and the EuroQol Visual Analogue Scale [EQ-VAS]) was assessed in only 3 dimensions of the KDQOL.

### Measurement property assessment of the included studies

Structural validity, internal consistency, test–retest reliability, and hypothesis testing for construct validity were reported in the included studies. Measurement error and cross-cultural validity/measurement invariance, criterion validity and responsiveness were not reported in any study. The measurement properties of each study are in Table 4.

**Table 4:** Measurement properties reported in the included studies and summary of the results

**Author, year of publication**	**Bouidida et al. 2014** [26]	**Abd ElHafeez et al. 2012** [34]	**Gebrie et al. 2022** [33]	**Elamin et al. 2019** [25]	**Bagasha et al. 2022** [35]
Structural validity	NR	**(+)**	**(+)**	NR	NR
		KMO = 0.73 Bartlett’s test: *p* < 0.001	RMSEA = 0.085[0.064–0.095]; CFI = 0.854; TLI = 0.838; SRMR = 0.067		
Internal consistency	(-)	(-)	**(+)**	(-)	**(-)**
	Cronbach’s alpha = 0.38–0.89	Cronbach’s alpha = 0.23–0.95	Cronbach’s alpha = 0.81–0.91	Cronbach’s alpha = 0.66–0.86	Cronbach’s alpha = 0.41–0.96
Test–retest reliability	(-)	**(+)**	**(+)**	**(+)**	NR
	ICC = 0.67–0.90	ICC = 0.79–0.95	ICC = 0.90–0.96	ICC = 0.74–0.98	
Hypotheses testing for construct validity	(-)	(-)	**(+)**	(-)	**(-)**

Measurement error, cross-cultural validity, criterion validity, and responsiveness were not reported in any of the 5 studies

( +) sufficient, (–) insufficient, (?) indeterminate, *NR* not reported, *KMO* Kaiser–Meyer–Olkin, *ICC* intraclass correlation coefficient, *CFI* comparative fit index, *RMSEA* root mean square error of approximation, *SRMR* standardized root mean residuals, *TLI* Tucker‐Lewis index

### Reliability

*Internal consistency*: One study was rated sufficient [33] and 4 were rated insufficient because the subscale values of Cronbach’s alpha were < 0.70 [25, 26, 34, 35].

*Test–retest reliability*: Test–retest reliability was assessed with the ICC in 4 studies [25, 26, 33, 34]: it was rated sufficient in 3 studies [25, 33, 34] and insufficient in 1 because of ICC < 0.7 [26].

### Validity

*Structural validity* was assessed with confirmatory factor analysis in 1 study [33] and exploratory factor analysis in 1 study [34]. It was rated sufficient in these 2 studies because of standardized root mean squared residual < 0.08 [33] and the Kaiser–Meyer–Olkin (KMO) was above the recommended value of 0.60 [34].

*Hypothesis testing for construct validity*: This was rated insufficient in 4 studies [25, 26, 34, 35] because the comparator had insufficient measurement properties or no information on these measurement properties. It was rated sufficient in 1 study [33]. The correlation of scores for 3 sub-dimensions of the KDQOL-36 (symptoms/problem, effect and burden of kidney disease) with those of comparator instruments (EQ-5D-5L and EQ-VAS) was > 0.5 [33].

## Discussion

This is the first systematic review to evaluate the psychometric properties of HRQoL questionnaires used in African patients with CKD by using the COSMIN checklist. We found a small number of studies of cultural adaptation and psychometric properties of an HRQoL questionnaire in African populations. All studies used the KDQOL questionnaire. The KDQOL-36, the most frequently used PROM in nephrology and adapted in several populations, consist of 5 dimensions, 2 are generic (“*physical component summary”* and “*mental component summary*”) and 3 are specific to kidney disease, including the “*symptoms and problems lists*”, “*effect of kidney disease*” and “*burden of kidney disease*”. The five dimensions are summarized by a score ranging from 0 to 100 (the lower the score, the more impaired HRQoL). These studies were generally of poor methodological quality according to the COSMIN checklist. Many psychometric properties, such as PROM development, structural validity, measurement error, cross-cultural validity, criterion validity, and responsiveness, were not reported in these studies.

In line with our findings, Aiyegbusi et al. [17] and Yangoz et al. [18], reported a small number of studies of cultural adaptation and psychometric properties of the KDQOL in African populations. Yet, several studies have assessed the HRQoL of African CKD patients with this questionnaire [15, 17, 36–41]. In this context, does this questionnaire really measure what it is supposed to measure? Is this questionnaire culturally adapted to the context of Africa? If any benefit is to be derived from the use of PROMs, they must actually measure what they are intended to measure (validation), produce consistent results (reliability), and capture all aspects of the construct(s) being studied that matter to the target population (adaptation) [18, 42]. African researchers must first validate and culturally adapt these questionnaires in their context before using them to obtain a reliable assessment of HRQoL in CKD. For longitudinal studies, responsiveness must be assessed in validation and cultural adaptation studies.

Good quality assessment of the consequences of CKD and the disability, like HRQoL, provides a better understanding of patient’s experience and the impact of their chronic disease and comorbidities, a better consideration of these consequences, an outcome of interest in clinical research and a better management, beyond the management of biological symptoms.

According to COSMIN guidelines, we found several methodological issues with the HRQoL questionnaire assessed in this review: no information on the clinical course of the patients or the time interval in test–retest reliability, failure to perform confirmatory factor analysis for construct validity, small sample sizes, unspecified missing data, lack of clear hypotheses for construct validity, no information on the psychometric properties of the comparators in construct validity, and no information on measurement error and responsiveness. Similar issues were reported by Aiyegbusi et al. [18]. A limitation of the evaluation of the methodological quality with COSMIN is an element of subjectivity because particular appreciations are left to the raters.

Despite these methodological issues, the KDQOL may have a potential utility in clinical practice in some African countries. The use of an HRQoL questionnaire in clinical practice has several benefits, including enhancing the communication between the patient and the clinician [43], facilitating reporting of serious adverse events [44], helping renal teams with the development of strategies to improve the HRQoL of CKD patients [45], and integrating routinely collected clinical and laboratory data (big PRO data) with several opportunities in patient care, population health management, and research [46]. Routine collection of HRQoL may allow for better management of quality of life including symptoms. These findings confirm the need to plan similar studies, but with adaptation of the questionnaire to the cultural context of the population, in order to have a better vision of the different aspects of the HRQoL of the patient with CKD and with these elements to outline a strategy to improve the condition of the patients. African researchers have a lot of work to do in terms of research, and must necessarily equip themselves with good instruments for assessment HRQoL.

The main strength of this systematic review is the use of the international COSMIN guidelines [29]. The use of PubMed/MEDLINE filters to build the search equation is also of value [31]. However, the study also has limitations. First, the included studies did not adequately report their assessments for a number of measurement properties. Second, because of the same questionnaire (KDQOL) studied in all studies and the small number of studies, we could not summarize the evidence and grade the quality of the evidence by using the GRADE approach recommended by COSMIN.

## Conclusion

Our review highlighted the publication of only a small number of HRQoL questionnaire validation studies, all with KDQOL, in African CKD populations. These studies are of poor methodological quality according to the COSMIN checklist. HRQoL validation studies in African CKD populations are needed to better take advantage of the benefits of such outcome in patient care, population health management, and research.

### Supplementary Information

**Supplementary file 1: Additional file 1.** PRISMA 2020 for Abstracts Checklist. **Additional file 2.** PRISMA checklist. **Additional file 3.** Search equations.

## Data Availability

All data generated or analysed during this study are included in this published article [and its supplementary information files].

## Abbreviations

COSMIN: COnsensus-based Standards for the selection of health status Measurement INstruments
HRQoL: Health-Related Quality of Life
CKD: Chronic Kidney Disease
PROM: Patient-Reported Outcome Measures
KDQOL: Kidney Disease Quality of Life
PRISMA: Preferred Reporting Items for Systematic Reviews and Meta-Analysis
ICC: Intraclass Correlation
DASS-21: Depression Anxiety Stress Scale-21
EQ-5D-5L: 5-Level EuroQol 5-dimensional questionnaire
EQ-VAS: EuroQol Visual Analogue Scale
