# Supplementary material for: Quality-of-life measures and their psychometric properties used in African chronic kidney disease populations: a systematic review using COSMIN methodology
Source: BMC Nephrol. 2024 Feb 9;25:50. doi: 10.1186/s12882-024-03482-5 (PMC10854046; doi:10.1186/s12882-024-03482-5)
Supplement: Supplementary file 1 — Additional file 1. PRISMA 2020 for Abstracts Checklist. Additional file 2. PRISMA checklist. Additional file 3. Search equations. [file 12882_2024_3482_MOESM1_ESM.pdf]

Additional files

Additional File 1: RISMA 2020 for Abstracts Checklist

Section and Topic	Item #	Checklist item	Reported (Yes/No)
**TITLE**			
Title	1	Identify the report as a systematic review.	Yes
**BACKGROUND**			
Objectives	2	Provide an explicit statement of the main objective(s) or question(s) the review addresses.	Yes
**METHODS**			
Eligibility criteria	3	Specify the inclusion and exclusion criteria for the review.	Yes
Information sources	4	Specify the information sources (e.g. databases, registers) used to identify studies and the date when each was last searched.	Yes
Risk of bias	5	Specify the methods used to assess risk of bias in the included studies.	Yes
Synthesis of results	6	Specify the methods used to present and synthesise results.	Not applicable
**RESULTS**			
Included studies	7	Give the total number of included studies and participants and summarise relevant characteristics of studies.	Yes
Synthesis of results	8	Present results for main outcomes, preferably indicating the number of included studies and participants for each. If meta-analysis was done, report the summary estimate and confidence/credible interval. If comparing groups, indicate the direction of the effect (i.e. which group is favoured).	Yes
**DISCUSSION**			
Limitations of evidence	9	Provide a brief summary of the limitations of the evidence included in the review (e.g. study risk of bias, inconsistency and imprecision).	Yes
Interpretation	10	Provide a general interpretation of the results and important implications.	Yes
**OTHER**			
Funding	11	Specify the primary source of funding for the review.	No

Section and Topic	Item #	Checklist item	Reported (Yes/No)
Registration	12	Provide the register name and registration number.	No

Additional File 2: PRISMA checklist

Section and Topic	Item #	Checklist item	Location where item is reported
**TITLE**			
Title	1	Identify the report as a systematic review.	P1, line 2
**ABSTRACT**			
Abstract	2	See the PRISMA 2020 for Abstracts checklist.	P2, line 15-37
**INTRODUCTION**			
Rationale	3	Describe the rationale for the review in the context of existing knowledge.	P3,line 40-79
Objectives	4	Provide an explicit statement of the objective(s) or question(s) the review addresses.	P4, line 79-82
**METHODS**			
Eligibility criteria	5	Specify the inclusion and exclusion criteria for the review and how studies were grouped for the syntheses.	P5, line 101-106
Information sources	6	Specify all databases, registers, websites, organisations, reference lists and other sources searched or consulted to identify studies. Specify the date when each source was last searched or consulted.	P5, line 92-99
Search strategy	7	Present the full search strategies for all databases, registers and websites, including any filters and limits used.	Additional file 2
Selection process	8	Specify the methods used to decide whether a study met the inclusion criteria of the review, including how many reviewers screened each record and each report retrieved, whether they worked independently, and if applicable, details of automation tools used in the process.	P6, line 107-110
Data collection process	9	Specify the methods used to collect data from reports, including how many reviewers collected data from each report, whether they worked independently, any processes for obtaining or confirming data from study investigators, and if applicable, details of automation tools used in the process.	P6, line 129
Data items	10a	List and define all outcomes for which data were sought. Specify whether all results that were compatible with each outcome domain in each study were sought (e.g. for all measures, time points, analyses), and if not, the methods used to decide which results to collect.	P6, line 121-127
	10b	List and define all other variables for which data were sought (e.g. participant and intervention characteristics, funding sources). Describe any assumptions made about any missing or unclear information.	P6, line129-131
Study risk of bias	11	Specify the methods used to assess risk of bias in the included studies, including details of the tool(s) used, how many reviewers assessed each	P6, line

Section and Topic	Item #	Checklist item	Location where item is reported
assessment		study and whether they worked independently, and if applicable, details of automation tools used in the process.	111-120
Effect measures	12	Specify for each outcome the effect measure(s) (e.g. risk ratio, mean difference) used in the synthesis or presentation of results.	Not adapted
Synthesis methods	13a	Describe the processes used to decide which studies were eligible for each synthesis (e.g. tabulating the study intervention characteristics and comparing against the planned groups for each synthesis (item #5)).	Not adapted
	13b	Describe any methods required to prepare the data for presentation or synthesis, such as handling of missing summary statistics, or data conversions.	Not adapted
	13c	Describe any methods used to tabulate or visually display results of individual studies and syntheses.	Not adapted
	13d	Describe any methods used to synthesize results and provide a rationale for the choice(s). If meta-analysis was performed, describe the model(s), method(s) to identify the presence and extent of statistical heterogeneity, and software package(s) used.	Not adapted
	13e	Describe any methods used to explore possible causes of heterogeneity among study results (e.g. subgroup analysis, meta-regression).	Not adapted
	13f	Describe any sensitivity analyses conducted to assess robustness of the synthesized results.	Not adapted
Reporting bias assessment	14	Describe any methods used to assess risk of bias due to missing results in a synthesis (arising from reporting biases).	
Certainty assessment	15	Describe any methods used to assess certainty (or confidence) in the body of evidence for an outcome.	Not adapted
**RESULTS**			
Study selection	16a	Describe the results of the search and selection process, from the number of records identified in the search to the number of studies included in the review, ideally using a flow diagram.	P7, line 137-140; figure 1
	16b	Cite studies that might appear to meet the inclusion criteria, but which were excluded, and explain why they were excluded.	P7, lines 142-171, Figure 1
Study characteristics	17	Cite each included study and present its characteristics.	P8, line 172-181; table 2
Risk of bias in studies	18	Present assessments of risk of bias for each included study.	P9 et P10, line 199-225; table 3
Results of individual studies	19	For all outcomes, present, for each study: (a) summary statistics for each group (where appropriate) and (b) an effect estimate and its precision (e.g. confidence/credible interval), ideally using structured tables or plots.	P11 et P12, line 228-250; table 4
Results of syntheses	20a	For each synthesis, briefly summarise the characteristics and risk of bias among contributing studies.	Not adapted
	20b	Present results of all statistical syntheses conducted. If meta-analysis was done, present for each the summary estimate and its precision (e.g.	Not adapted

Section and Topic	Item #	Checklist item	Location where item is reported
		confidence/credible interval) and measures of statistical heterogeneity. If comparing groups, describe the direction of the effect.	
	20c	Present results of all investigations of possible causes of heterogeneity among study results.	Not adapted
	20d	Present results of all sensitivity analyses conducted to assess the robustness of the synthesized results.	Not adapted
Reporting biases	21	Present assessments of risk of bias due to missing results (arising from reporting biases) for each synthesis assessed.	Not adapted
Certainty of evidence	22	Present assessments of certainty (or confidence) in the body of evidence for each outcome assessed.	Not adapted
**DISCUSSION**			
Discussion	23a	Provide a general interpretation of the results in the context of other evidence.	P12, line 277-305
	23b	Discuss any limitations of the evidence included in the review.	P15, line 322-323
	23c	Discuss any limitations of the review processes used.	P15, line 323-326
	23d	Discuss implications of the results for practice, policy, and future research.	P14-15, line 306-319
**OTHER INFORMATION**			
Registration and protocol	24a	Provide registration information for the review, including register name and registration number, or state that the review was not registered.	P5, line 90-91
	24b	Indicate where the review protocol can be accessed, or state that a protocol was not prepared.	P5, line 90-91
	24c	Describe and explain any amendments to information provided at registration or in the protocol.	
Support	25	Describe sources of financial or non-financial support for the review, and the role of the funders or sponsors in the review.	P16, line 360
Competing interests	26	Declare any competing interests of review authors.	P16, line 359
Availability of data, code and other materials	27	Report which of the following are publicly available and where they can be found: template data collection forms; data extracted from included studies; data used for all analyses; analytic code; any other materials used in the review.	P16, line 356

From: Page MJ, McKenzie JE, Bossuyt PM, Boutron I, Hoffmann TC, Mulrow CD, et al. The PRISMA 2020 statement: an updated guideline for reporting systematic reviews. BMJ 2021;372:n71. doi: 10.1136/bmj.n71

For more information, visit: http://www.prisma-statement.org/

Additional File 2

PubMed search equation

(instrumentation[sh] OR methods[sh] OR Validation Studies[pt] OR Comparative Study[pt] OR ‘‘psychometrics’’ [MeSH] OR psychometr*[tiab] OR clinimetr*[tw] OR clinometr*[tw] OR ‘‘outcome assessment (health care)’’[MeSH] OR outcome assessment[tiab] OR outcome measure*[tw] OR ‘‘observer variation’’[MeSH] OR observer variation[tiab] OR ‘‘Health Status Indicators’’[Mesh] OR ‘‘reproducibility of results’’[MeSH] OR reproducib*[tiab] OR ‘‘discriminant analysis’’[MeSH] OR reliab*[tiab] OR unreliab*[tiab] OR valid*[tiab] OR coefficient[tiab] OR homogeneity[tiab] OR homogeneous[tiab] OR ‘‘internal consistency’’[tiab] OR (cronbach*[tiab] AND (alpha[tiab] OR alphas[tiab])) OR (item[tiab] AND (correlation*[tiab] OR selection*[tiab] OR reduction*[tiab])) OR agreement[tiab] OR precision[tiab] OR imprecision[tiab] OR ‘‘precise values’’[tiab] OR testretest[tiab] OR (test[tiab] AND retest[tiab]) OR (reliab* [tiab] AND (test[tiab] OR retest[tiab])) OR stability[tiab] OR interrater[tiab] OR inter-rater[tiab] OR intrarater[tiab] OR intra-rater[tiab] OR intertester[tiab] OR inter-tester[tiab] OR intratester[tiab] OR intra-tester[tiab] OR interobserver[tiab] OR inter-observer[tiab] OR intraobserver[tiab] OR intraobserver[tiab] OR intertechnician[tiab] OR inter-technician[tiab] OR intratechnician[tiab] OR intra-technician[tiab] OR interexaminer[tiab] OR inter-examiner[tiab] OR intraexaminer[tiab] OR intra-examiner[tiab] OR interassay[tiab] OR inter-assay[tiab] OR intraassay[tiab] OR intra-assay[tiab] OR interindividual[tiab] OR inter-individual[tiab] OR intraindividual[tiab] OR intra-individual[tiab] OR interparticipant [tiab] OR inter-participant[tiab] OR intraparticipant[tiab]

OR intra-participant[tiab] OR kappa[tiab] OR kappa's[tiab] OR kappas[tiab] OR repeatab*[tiab] OR ((replicab*[tiab] OR repeated[tiab]) AND (measure[tiab] OR measures[tiab] OR findings[tiab] OR result[tiab] OR results[tiab] OR test[tiab] OR tests[tiab])) OR generaliza*[tiab] OR generalisa*[tiab] OR concordance[tiab] OR (intraclass[tiab] AND correlation*[tiab]) OR discriminative[tiab] OR ''known group''[tiab] OR factor analysis[tiab] OR factor analyses[tiab] OR dimension*[tiab] OR subscale*[tiab] OR (multitrait[tiab] AND scaling[tiab] AND (analysis[tiab] OR analyses[tiab])) OR item discriminant[tiab] OR interscale correlation*[tiab] OR error[tiab] OR errors[tiab] OR ''individual variability''[tiab] OR (variability[tiab] AND (analysis[tiab] OR values[tiab])) OR (uncertainty[tiab] AND (measurement[tiab] OR measuring[tiab])) OR ''standard error of measurement''[tiab] OR sensitiv*[tiab] OR responsive*[tiab] OR ((minimal[tiab] OR minimally[tiab] OR clinical[tiab] OR clinically[tiab]) AND (important[tiab] OR significant[tiab] OR detectable[tiab]) AND (change[tiab] OR difference[tiab])) OR (small*[tiab] AND (real[tiab] OR detectable[tiab]) AND (change[tiab] OR difference[tiab])) OR meaningful change [tiab] OR ''ceiling effect''[tiab] OR ''floor effect''[tiab] OR ''Item response model''[tiab] OR IRT[tiab] OR Rasch[tiab] OR ''Differential item functioning''[tiab] OR DIF[tiab] OR ''computer adaptive testing''[tiab] OR ''item bank''[tiab] OR ''cross-cultural equivalence''[tiab]) AND ("chronic kidney disease" OR "Kidney Failure, Chronic"[Mesh] OR "Renal Insufficiency, Chronic"[Mesh] OR "Peritoneal Dialysis"[Mesh] OR "Hemodialysis, Home"[Mesh] OR "Renal Dialysis"[Mesh] OR "Dialysis"[Mesh] OR "Peritoneal Dialysis, Continuous Ambulatory"[Mesh] OR "Hemodialysis Units, Hospital"[Mesh] OR "hemodialysis") AND ('quality of life questionnaire' OR 'quality of life scale' OR 'health related quality of life questionnaire' OR 'kidney disease quality of life short form'/exp OR 'kidney disease quality of life' OR 'kidney disease quality of life 36' OR 'kidney disease quality of life questionnaire' OR 'dialysis symptom index' OR 'edmonton symptom assessment system' OR 'whoqol-100' OR 'whoqol-bref' OR 'quality of life

index’ OR ‘nottingham health profile’ OR ‘nottingham health profile questionnaire’ OR ‘short form 36’ OR ‘short form 12’) AND (“Algeria” OR “Angola” OR “Benin” OR “Botswana” OR “Burkina Faso” OR “Burundi” OR “Cabo Verde” OR “Cameroon” OR “Central African Republic” OR “Chad“ OR “Comoros“ OR “Congo“ OR “Democratic Republic of the Congo“ OR “Cote d’Ivoire“ OR “Djibouti“ OR “Egypt“ OR “Equatorial Guinea“ OR “Eritrea“ OR “Eswatini“ OR “Ethiopia“ OR “Gabon“ OR “Gambia“ OR “Ghana“ OR “Guinea“ OR “Guinea-Bissau“ OR “Kenya“ OR “Lesotho“ OR “Liberia“ OR “Libya“ OR “Madagascar“ OR “Malawi“ OR “Mali“ OR “Mauritania“ OR “Mauritius“ OR “Morocco“ OR “Mozambique“ OR “Namibia“ OR “Niger“ OR “Nigeria“ OR “Rwanda“ OR “Sao Tome and Principe“ OR “Senegal“ OR “Seychelles“ OR “Sierra Leone“ OR “Somalia“ OR “South Africa“ OR “South Sudan“ OR “Sudan“ OR “Tanzania“ OR “Togo“ OR “Tunisia“ OR “Uganda“ OR “Zambia“ OR “Zimbabwe“)

Embase search equation

(‘chronic kidney failure’/exp OR ‘chronic kidney disease’ OR ‘chronic kidney disorder’ OR ‘chronic kidney failure’ OR ‘chronic kidney insufficiency’ OR ‘chronic nephropathy’ OR ‘chronic renal disease’ OR ‘chronic renal failure’ OR ‘chronic renal insufficiency’ OR ‘kidney chronic failure’ OR ‘kidney disease, chronic’ OR ‘kidney failure, chronic’ OR ‘kidney function, chronic disease’ OR ‘renal insufficiency, chronic’ OR ‘dialysis’/exp OR ‘renal replacement therapy’/exp OR ‘hemodialysis’/exp OR ‘peritoneal dialysis’/exp OR ‘kidney transplantation’/exp OR ‘kidney graft’/exp) **AND (**‘quality of life questionnaire’/exp OR ‘quality of life scale’/exp OR ‘health related quality of life questionnaire’/exp OR ‘kidney disease quality of life short form’/exp OR ‘kidney disease quality of life’/exp OR ‘kidney disease quality of life 36’/exp OR ‘kidney disease quality of life questionnaire’/exp OR ‘dialysis symptom index’/exp OR ‘edmonton symptom assessment system’/exp OR ‘whoqol-100’/exp OR ‘whoqol-bref’/exp OR ‘quality of life index’/exp OR ‘nottingham health profile’/exp OR ‘nottingham health profile questionnaire’/exp OR ‘short form 36’/exp OR ‘short form 12’/exp) **AND (**‘psychometry’/exp OR ‘validation study’/exp OR ‘instrumentation recorder’/exp OR ‘comparative study’/exp OR ‘reproducibility’/exp OR ‘observer variation’/exp OR ‘discriminant analysis’/exp OR ‘reliability’/exp OR ‘validity’/exp OR ‘homogeneity’/exp OR ‘Cronbach alpha coefficient’/exp OR ‘alpha’/exp OR ‘item analysis’/exp OR ‘item’/exp OR ‘item total correlation’/exp OR ‘item response theory’/exp OR ‘item response theory analysis’/exp OR ‘agreement’/exp OR ‘correlation coefficient’/exp OR ‘correlation analysis’/exp OR ‘test’/exp OR ‘interrater reliability’/exp OR ‘intrarater reliability’/exp OR ‘intertester reliability’/exp OR ‘interobserver

variability'/exp OR 'interobserver variation'/exp OR 'interobserver reproducibility'/exp OR 'interindividual variability'/exp OR 'interindividual variation'/exp OR 'intraindividual variability'/exp OR 'kappa statistics'/exp OR 'repeatability'/exp OR 'replicability'/exp OR 'measurement'/exp OR 'measurement repeatability'/exp OR 'measurement error'/exp OR 'measurement precision'/exp OR 'concordance correlation coefficient'/exp OR 'concordance'/exp OR 'known group validity'/exp OR 'known groups validity'/exp OR 'factor analysis'/exp OR 'dimension'/exp OR 'scale'/exp OR 'multitrait multimethod'/exp OR 'scaling'/exp OR 'analysis'/exp OR 'error'/exp OR 'variability'/exp OR 'uncertainty'/exp OR 'standard error of measurement'/exp OR 'sensitivity and specificity'/exp OR 'sensitivity and sensibility'/exp OR 'responsiveness'/exp OR 'change'/exp OR 'ceiling effect'/exp OR 'floor effect'/exp OR 'Rasch analysis'/exp OR 'differential item functioning'/exp OR 'differential item functioning analysis'/exp OR 'computer adaptive testing'/exp OR 'cross cultural'/exp OR 'cross cultural adaptation'/exp OR 'cross cultural validity'/exp)

Web of Science search equation

((((ALL=(chronic kidney failure OR chronic kidney disease OR chronic kidney disorder OR chronic kidney failure OR chronic kidney insufficiency OR chronic nephropathy OR chronic renal disease OR chronic renal failure OR chronic renal insufficiency OR kidney chronic failure OR dialysis OR renal replacement therapy OR hemodialysis OR peritoneal dialysis OR kidney transplantation OR kidney graft)) AND ALL=(quality of life OR quality of life questionnaire)) AND ALL=(Africa)) AND TS=(psychomet* OR ‘validation study OR valid* OR instrumentation recorder OR comparative study OR reproducibility OR observer variation OR discriminant analysis OR reliability OR validity OR homogeneity OR crenbach alpha coefficient OR alpha OR item analysis OR item OR item total correlation OR item response theory OR item response theory analysis OR agreement OR correlation coefficient OR correlation analysis OR test OR integrater reliability OR intralayer reliability OR interester reliability OR interserver variability OR interserver variation OR interserver reproducibility OR interindividual variability OR interindividual variation OR interindividual variability OR kappa statistics OR repeatability OR replicability OR measurement OR measurement repeatability OR measurement error OR measurement precision OR concordance correlation coefficient OR concordance OR known group validity OR known groups validity OR factor analysis OR dimension OR scale OR multitrain multimethod OR scaling OR analysis OR error OR variability OR uncertainty OR standard error of measurement OR sensitivity and specificity OR sensitivity and sensibility OR responsiveness OR change OR ceiling effect OR

floor effect OR ranch analysis OR differential item functioning OR differential item functioning analysis OR computer adaptive testing OR cross cultural OR cross cultural adaptation OR cross cultural validity))

PsycINFO search equation

(chronic kidney failure OR chronic kidney disease OR chronic kidney disorder OR chronic kidney failure OR chronic kidney insufficiency OR chronic nephropathy OR chronic renal disease OR chronic renal failure OR chronic renal insufficiency OR kidney chronic failure OR dialysis OR renal replacement therapy OR hemodialysis OR peritoneal dialysis OR kidney transplantation OR kidney graft) AND (quality of life questionnaire OR quality of life scale OR health related quality of life questionnaire OR kidney disease quality of life short form OR kidney disease quality of life OR kidney disease quality of life 36 OR kidney disease quality of life questionnaire OR dialysis symptom index OR edmonton symptom assessment system OR whoqol-100 OR whoqol-bref OR quality of life index OR nottingham health profile OR nottingham health profile questionnaire OR short form 36 OR short form 12) AND (psychomet* OR ‘validation study OT valid* OR instrumentation recorder OR comparative study OR reproducibility OR observer variation OR discriminant analysis OR reliability OR validity OR homogeneity OR crenbach alpha coefficient OR alpha OR item analysis OR item OR item total correlation OR item response theory OR item response theory analysis OR agreement OR correlation coefficient OR correlation analysis OR test OR integrater reliability OR intralayer reliability OR interester reliability OR interserver variability OR interserver variation OR interserver reproducibility OR interindividual variability OR interindividual variation OR interindividual variability OR kappa statistics OR repeatability OR replicability OR measurement OR measurement repeatability OR measurement

error OR measurement precision OR concordance correlation coefficient OR concordance OR known group validity OR known groups validity OR factor analysis OR dimension OR scale OR multitrain multimethod OR scaling OR analysis OR error OR variability OR uncertainty OR standard error of measurement OR sensitivity and specificity OR sensitivity and sensibility OR responsiveness OR change OR ceiling effect OR floor effect OR ranch analysis OR differential item functioning OR differential item functioning analysis OR computer adaptive testing OR cross cultural OR cross cultural adaptation OR cross cultural validity) AND (africa)
